# Monitoring the impact of trade agreements on national food environments: trade imports and population nutrition risks in Fiji

**DOI:** 10.1186/s12992-017-0257-1

**Published:** 2017-06-13

**Authors:** Amerita Ravuvu, Sharon Friel, Anne-Marie Thow, Wendy Snowdon, Jillian Wate

**Affiliations:** 1 School of Regulation and Global Governance (RegNet), The Australian National University, H.C. Coombs Extension Building #8, Fellows Road ACT, Canberra, 0200 Australia; 2 Menzies Centre for Health Policy, School of Public Health, The University of Sydney, Sydney, Australia; 3 Centre for Population Health Research, Deakin University, Burwood, Australia; 4 Pacific Research Centre for the Prevention of Obesity and Non-Communicable Diseases (C-POND), College of Medicine, Nursing and Health Sciences, Fiji National University, Suva, Fiji

**Keywords:** Trade agreements, Monitoring, Food environments, Food imports, Foreign investment

## Abstract

**Background:**

Trade agreements are increasingly recognised as playing an influential role in shaping national food environments and the availability and nutritional quality of the food supply. Global monitoring of food environments and trade policies can strengthen the evidence base for the impact of trade policy on nutrition, and support improved policy coherence. Using the INFORMAS trade monitoring protocol, we reviewed available food supply data to understand associations between Fiji’s commitments under WTO trade agreements and food import volume trends.

**Methods:**

First, a desk review was conducted to map and record in one place Fiji’s commitments to relevant existing trade agreements that have implications for Fiji’s national food environment under the domains of the INFORMAS trade monitoring protocol. An excel database was developed to document the agreements and their provisions. The second aspect of the research focused on data extraction. We began with identifying food import volumes into Fiji by country of origin, with a particular focus on a select number of ‘healthy and unhealthy’ foods. We also developed a detailed listing of transnational food corporations currently operating in Fiji.

**Results:**

The study suggests that Fiji’s WTO membership, in conjunction with associated economic and agricultural policy changes have contributed to increased availability of both healthy and less healthy imported foods. In systematically monitoring the import volume trends of these two categories of food, the study highlights an increase in healthy foods such as fresh fruits and vegetables and whole-grain refined cereals. The study also shows that there has been an increase in less healthy foods including fats and oils; meat; processed dairy products; energy-dense beverages; and processed and packaged foods.

**Conclusion:**

By monitoring the trends of imported foods at country level from the perspective of trade agreements, we are able to develop appropriate and targeted interventions to improve diets and health. This would enable national health interventions to both identify areas of concern, and to ensure that interventions take into account the trade context.

## Background

There is increasing evidence internationally of the relationship between trade agreements (see Table 1 for definition of trade-related terminology), changes in food environments and the increases in levels of obesity and diet-related non-communicable diseases [1–7]. Trade policies which emphasise greater market access for food exports and opening of domestic markets for foreign investment have facilitated the creation of food environments full of processed foods, which are often high in fat, salt and sugar [8]. A systematic approach to monitoring the impact of trade agreements on the food supply at country level is an essential basis for developing appropriate and targeted interventions to improve diets and health. This would enable national health interventions to both identify areas of concern, and to ensure that interventions take into account the trade context.

**Table 1:** Glossary of trade policy terminology and definitions

Anti-dumping	WTO Agreement disciplining anti-dumping actions that governments may use to react to products being ‘dumped’ into their domestic market.
Customs valuation	Customs procedure applied to determine the customs value of imports.
Foreign direct investment (FDI)	An investment in a country other than that of the investor, involving a long-term relationship and substantial, but not necessarily majority, interest in an enterprise by the investor. Foreign direct investment can take place through direct entry or investment in existing firms.
General Agreement on Trade in Services (GATS)	WTO agreement governing services trade. Requires member countries to provide national treatment to foreign service providers in those service industries that they have agreed to liberalise under GATS.
General Agreement on Tariffs and Trade (GATT)	Multilateral FTA first signed in 1947 between 23 countries. Superceded by the WTO in 1995. Updated GATT (1994) is now one of the WTO’s agreements.
Import licensing	Controls imposed by the state on importers.
National treatment (NT)	The principle of giving foreign firms ‘no less favourable’ treatment than domestic firms/goods once border measures have been applied. Internal tax and regulatory measures must be applied equally to imported and domestic goods or committed (scheduled) services in order to avoid trade disputes.
Non-tariff measures/barriers to trade	Government measures other than tariffs that restrict trade flows (e.g. quantitative restrictions on goods or services, import licensing, variable levies, import barriers and Technical Barriers to Trade.
Policy space	The freedom, scope and mechanisms that governments have to choose, design and implement public policies to fulfil their aims.
Pre-shipment Inspection	WTO Agreement focussing on quality control to check shipment details such as price, quantity and quality of goods ordered overseas.
Rules of Origin (ROO)	Criteria needed to determine the national source of a product. ROO is particularly important for the enforcement of duties and restrictions depending on the source of products.
Sanitary and phytosanitary (SPS) measures	Technical barriers designed for the protection of human health or the control of animal and plant pests and diseases.
Subsidies and Countervailing Measures/Safeguards	WTO member countries agreed to the use of disciplinary measures against subsidies and this agreement also regulates actions WTO member countries can take to counter the effects of subsidies.
Tariffs, applied tariffs/rates, and bound tariffs/rates	An applied tariff/rate is a custom duty (tax) applied on imported goods at the border. Tariffs are levied either on an ad valorem basis (percentage of value) or on a specific basis (e.g. by weight or volume). Bound tariffs/rates are enforceable and are the highest rate that a WTO member country can charge on imports without attracting an appeal for compensation by the affected country. For this reason, tariffs/rates actually applied on imports are typically lower than bound tariffs/rates.
Technical Barriers to Trade (TBT)	Non-tariff regulations, standards, testing and certification procedures, which can create obstacles to trade. WTO member countries agreed to the use of disciplinary measures against TBTs on both industrial and agricultural products as part of the Uruguay Round of multilateral trade negotiations (1986–1993).
Trade agreement	A negotiated agreement between two or more countries to limit or alter their policies with respect to trade. Trade agreements can be bilateral, regional or multilateral.
Trade liberalization	The reduction or removal of barriers to trade in order to create a ‘free’ market in goods, services or finance.
TRIMs	The WTO’s Agreement on Trade-Related Investment Measures, which requires member countries to phase out (and refrain from implementing) trade distorting or restricting investment measures that are inconsistent with GATT principles.
TRIPS	The WTO’s Agreement on Trade-Related Aspects of Intellectual Property Rights which stipulates minimum standards of intellectual property protection.
World Trade Organization	Replaced the GATT in 1995 as the legal and institutional foundation of the multilateral trading system of member countries following the Uruguay Round.

Adapted from Friel et al., 2013 [15]

As Small Island Developing States, Pacific island countries (PICs) are particularly vulnerable to the processes of globalisation, which emphasise trade and investment liberalisation [9]. While trade has been viewed as a necessary development strategy to boost economic growth [9], trade liberalisation also appears to have created food environments across the PICs that are conducive to the widespread distribution of sugary, salty and fatty imported foods [10, 11]. Store surveys carried out in Fiji, Guam, Nauru, New Caledonia and Samoa identified 54 countries of origin for foods sold therein [12]. In Fiji, local food production has been largely impacted by free market policies [13] and previous research has shown that a majority of urban and rural populations in Fiji now rely on the cheapest imported foods such as white rice and noodles which have low nutritional quality [14]. While there is growing concern about the effect of trade measures on PIC populations’ nutrition and the levels of diet-related non-communicable diseases (NCDs) [6, 11], there appears to be no systematic monitoring of the impact of trade agreements on food environments and NCD risks.

In this paper we hypothesise that food availability, nutrition quality and the healthiness of Fiji’s food environment has changed due to the liberalisation of international trade and foreign direct investment (FDI) through multilateral trade agreements. The paper uses the International Network for Food and Obesity/NCDs Research, Monitoring and Action Support (INFORMAS) trade monitoring framework [15] to systematically examine changes in food import volumes coming to Fiji between 1980 and 2010 from all signatory countries to WTO agreements. INFORMAS is a global network of public-interest organisations and researchers that aims to monitor, benchmark, and support public and private sector actions to create healthy food environments and reduce obesity, NCDs and their related inequalities [16].

### Fiji trade environment

Fiji gained independence in 1970. As a response to the global oil shocks, progressive trade-restrictive and trade-protectionist measures were implemented to stabilise Fiji’s economy and protect its local industries [10]. An array of import tariffs, subsidies and quotas were placed to protect the rice, dairy, poultry, beef, pork and tobacco industries and to reduce competition from imported food. High import tariffs were placed on many processed foods [10]. With levels of public debt continuing to increase with the protectionist approach, the International Monetary Fund and the World Bank presented advice on a new economic policy direction based on the Washington Consensus, and by 1986 a new policy direction favouring an outward-looking export strategy was replacing the import substitution strategy [17]. Two military coups in 1987 increased this momentum [18] and the post-coup government continued to implement key reform measures that were less trade-restrictive and Fiji adopted a phased program to remove import licensing requirements and quantitative import restrictions, reduce tariffs, deregulate financial markets, reform the tax system, reform public enterprises and boost exports [17].

In 1993, Fiji became a member of the General Agreement on Tariffs and Trade. In accordance with Article XXVIII *bis* of GATT 1947, incorporated into the WTO GATT 1994 on tariff negotiations, the Article recognises that “customs duties often constitute serious obstacles to trade”. As a signatory of GATT and in its preparation towards WTO accession in 1996, the Government of Fiji undertook progressive tariff reforms in the early 1990s. For example, Fiji undertook a radical shift in its tax system, implementing the Value-Added Tax (VAT) in July 1992 and streamlined import duties [17]. From 1994 all import licensing controls on agricultural products (including all food products) were removed and these were replaced by tariffs [19]. Fiji chose a single bound rate of 40 per cent for all agricultural products, except for rice and milk powder [19].

Besides the removal of import licenses and the tariff reforms taking place, changes in food imports to Fiji was also accompanied by the restrictions imposed on non-tariff measures falling under the WTO provisions. Fiji is bound by non-tariff measures related to national treatment under Article III of GATT 1947 incorporated into Article III of GATT 1994. Article III of GATT 1994 deals with the principle of national treatment on imported goods with regard to internal taxations and regulations. The main obligation under Article III entails no internal taxes and other internal charges of any kind should be imposed on imported products in excess of those applied to like domestic products “so as to afford protection to domestic production”. The national treatment obligation under Article III calls for non-discrimination against imported goods.

Fiji became a member of the WTO in 1996. This means Fiji is part of the General Agreement on Trade in Services (GATS), General Agreement on Tariffs and Trade (GATT), the WTO Agreements on Agriculture (AOA), the Application of Sanitary and Phytosanitary Measures (SPS), Technical Barriers to Trade (TBT), Trade-Related Investment Measures (TRIMS), Anti-dumping, Customs Valuation, Preshipment Inspection, Rules of Origin, Import Licensing Procedures, Subsidies and Countervailing Measures, Safeguards, and Trade Related Aspects of Intellectual Property Rights agreement (TRIPS). Fiji is also a signatory to six regional agreements [20] detailed in Table 2.

**Table 2:** Fijian selected focus foods

Healthy foods		Less healthy foods	
Focus food category	Food product	Focus food category	Food product
Fresh Fruits	Citrus Fresh apples Fresh grapes	Edible oils and spreads	Palm Oil Corn Oil Hydrogenated fats Margarine Butter
Fresh vegetables	Fresh tomatoes Garlic Onion Leeks and other alliaceous vegetables Cauliflowers and broccoli Other cabbages and cauliflowers Cabbage lettuce Other lettuce, other vegetables Carrots and turnips Potatoes Celery (Beetroot, Radish)	Selected meat products	Chicken nuggets/patties Beef patties Canned foods (excluding fish)
Pulses, nuts and seeds	Dried leguminous vegetables, split, lentils, chickpeas and kidney beans	High processed dairy products	Processed cheese Fruit based/flavoured yoghurt Ice-cream and edible ices
Staple whole-grain cereals	Rice (brown) Rolled oats; oat meal Healthy breakfast cereals	Energy-dense beverages	Cordial juices (e.g. Cordial concentrate, tang, vita fresh powder, soft drink concentrate, Just Juice, Fruit drinks, Fruitace, Nutri-C) Sugar-sweetened, carbonated drinks Electrolyte drinks (e.g. Powerade, Gatorade, Red Bull, Mother, V-Drink)
		Sugar & other caloric sweeteners	Raw sugar/White Sugar/Refined/Manufactured, Sugar sachets, icing sugar, caster sugar
		Savoury ready to eat snacks & meals	Snack packs, corn chips, potato chips etc. Noodles (Flavoured, Instant)Confectionary
		Sweet snacks	Sweet biscuits
		Sweet packaged breakfast cereals	Highly processed & sugar sweetened (Coco Pops, Choco Puffs, Fruit flavoured, milo ball, fruit loops etc.)
		Whole-grain cereal	White rice

Adapted from: Friel et al., 2013 [1]; Legge et al., 2011 [24]; WTO Online database (www.wto.org) [29]; Pacific Islands Forum website (www.forumsec.org) [30, 31]; Fiji Revenue and Customs Authority website (http://www.frca.org.fj) [32] and the MSG Secretariat Online database (www.msgsec.info) [33]

In 1999, a new government took office and outlined a new programme of quantitative restrictions, increased tariffs and input subsidies to restore protection to the ailing rice industry and the agricultural sector as a whole. Changes in government policies resulting from a third and fourth military coup in the year 2000 and 2006 have since reintroduced import substitution measures and there have been relapses in tariff levels given that tariff remains a main trade policy instrument for Fiji [20].

In this study, we focus on the WTO agreements as all the other regional agreements reflected in Table 2 make only minor changes to Fiji’s commitments under the WTO, with respect to the national food supply. The associated food import volumes, tariffs and the type and country of origin of foreign investment from transnational food corporations coming to Fiji from all signatory countries to the WTO agreements during the study period is presented.

## Methods

The focus of the study is on the links between trade agreement provisions and food availability at the national level in Fiji. The INFORMAS trade monitoring framework and associated data collection protocol was used to guide the selection of indicators and analysis [15, 21].

### Selection of focus foods

A set of selected food categories rather than total food supply is recommended within the ‘minimal’ version of the INFORMAS trade monitoring framework. Suggested food categories on which to focus were identified from the literature and reflected the current Fijian diet [12, 22]. These are outlined in Table 3. Focus foods were identified and classified as ‘healthy’ or ‘less healthy’ based on the suggested focus food categories identified in Box 3 of the INFORMAS trade monitoring framework paper [15]. Specific food categories were selected based on Fiji data captured in a Store Survey conducted for the Pacific region [12]. The detailed methods of grouping focus foods into ‘healthy’ or ‘less healthy’ categories and the selection of specific foods for monitoring under these categories are outlined in the INFORMAS protocol paper [23].

**Table 3:** Key Fijian ratified trade agreements

Trade Agreement	Date Ratified	Partner Countries	Summary
South Pacific Regional Trade and Economic Cooperation Agreement (SPARTECA)	1981	Cook Island, Australia, Fiji, Marshall Islands, Micronesia, Nauru, New Zealand, Papua New Guinea, Samoa, Solomon Islands, Tonga, Tuvalu, Vanuatu, Kiribati, and Niue, Australia and New Zealand	Non-reciprocal regional trade agreement designed to allow Pacific Island countries progressive tariff-free access for many of their exports into the Australian and New Zealand markets.
Melanesian Spearhead Group Trade Agreement (MSGTA)	1998	Fiji, Vanuatu, Solomon Islands & Papua New Guinea	Preferential Free Trade Agreement (FTA) aimed at promoting regional integration, removing trade barriers and governing trade.
Pacific Island Countries Trade Agreement (PICTA)	2002	Cook Islands, Federated States of Micronesia, Fiji, Kiribati, Republic of the Marshall Islands, Nauru, Niue, Republic of Palau, Papua New Guinea, Samoa, Solomon Islands, Tonga, Tuvalu and Vanuatu	Regional Free Trade Agreement (FTA) governing trade in goods and aimed at establishing a single market by creating a free-trade area among the 14 Pacific island member countries.
Pacific Agreement on Closer Economic Relations (PACER)	2008	14 PICTA countries and Australia and New Zealand	Framework Agreement for future trade cooperation and economic integration between PICTA members and Australia and New Zealand aimed to create a single regional economy.
Cotonou Agreement	2003. This expired in 2007	Fiji, 79 developing countries in the Africa, Caribbean and Pacific region and the European Union	Partnership agreement governing development, political, economic and trade cooperation.
Interim Economic Partnership Agreement (iEPA)	2009	Fiji and the European Union	Following the expiration of the Cotonou Agreement in 2007, Fiji signed iEPA to protect its sugar exports to the European Community while EPA negotiations for preferential access to the EU market are currently being negotiated.

Adapted from Friel et al., 2013 [1] and Snowdon et al., 2013 [12]

### Food-related trade indicators

The INFORMAS monitoring framework comprises a set of indicators under four ‘domains’, each of which is informed by the international evidence relating to trade and nutrition: i) trade in goods; ii) trade in services and foreign direct investment (FDI); iii) domestic protections and supports; and iv) policy space. These are common elements of trade agreements that could have an impact on the healthfulness of food environments and have also been reviewed in the Pacific islands context presenting opportunities and risks for the prevention and management of NCDs [24].

In this analysis, we focus on the minimal monitoring approach in two of the domains: trade in goods, and trade in services and foreign direct investment. When preparing the INFORMAS protocol paper [23], we identified quite limited data availability in Fiji, particularly to do with FDI and domestic supports. The interrogation of policy space (domain 4) was also identified as being better captured using qualitative data methods. For this reason we focus now on trade in goods, and trade in services. Data was obtained for the following indicators relating to these two domains: total food import volumes; focus food category import volumes; actual and bound tariff rates for focus foods, and the type and country of origin of all foreign-owned transnational food corporations (TFCs) operating within Fiji. For total food import volumes, data were selected by food import categories as defined by the Fiji Bureau of Statistics (FBOS) and the specific classification codes used to classify these food items. In determining which ‘healthy’ and ‘less healthy’ foods to monitor for Fiji, the selection of ‘less healthy’ foods was based on the ‘Store Survey’ mentioned earlier and the decision of ‘healthy’ foods to collect data for was guided by information provided in the National Nutrition Survey (NNS) Reports undertaken for Fiji. The NNS Reports reflect dietary-intake patterns and the most common fresh fruits and vegetables found in the diet of the major ethnic groups. In the collection of actual and bound tariff rates, information for the year 2010 was collected from the WTO Tariff Download Facility^1^ while tariff rates for preceding years were collected from the Fiji Budget Summaries and WTO Trade Update Reports. Data collection concerning food-related foreign direct investment into Fiji was limited and data presented in this paper was collected through the International Trade Centre (ITC) database.^2^ The detailed methods of data collection for each indicator are outlined in the INFORMAS protocol paper [23].

Data on these indicators were analysed over time (using Microsoft Excel) and compared with changes in Fiji’s WTO commitments, to shed more light on the impact of trade. There were 112 WTO member countries exporting food into Fiji by 2010. In deciding the years to monitor food import volumes (i.e. 1980, 1990, 1996, 2000 and 2010), these were largely determined based on the escalation of imports prior to Fiji joining as a WTO member in 1996 and the trend of import volumes after the ratification and sign-off on the WTO agreement and the implementation phase. Additionally, these single points were used because of the limited data available for specific foods for the 1980 to 1999 period. Annual trade data from 1982 to 1989 was not readily available. Annual trade data from 1990 to 1999 was not segregated to reflect specific food categories that we were monitoring and only included major trading partners that Fiji traded with and not every country in the WTO. Detailed food import volume data is only now available for data dating back to 2005 as Fiji only implemented the Automated Systems of Customs Data (ASYCUDA) which captures and implements all international standards for trade data by specific categories in 2014. Consequently, we were unable to smooth data by using multiple year averages and had to select single points instead. Similarly, a comprehensive listing of information on Fiji’s actual and bound tariff rates for specific food categories is only available for the years 2006 to 2013 through the WTO Tariff Download Facility. Information on applied and bound tariffs for previous years were not readily available and as outlined in the protocol paper [23], Fiji Budget summaries and WTO Trade Update Reports were consulted for preceding years. Given the single points that we had selected to monitor food import volumes, in this paper we only reported tariff rates for these corresponding years i.e. 1990, 1996, 2000 and 2010. For the year 1980, there was no information on applied and bound tariffs for the specific food categories that we were monitoring, thus no tariff data is reported for 1980 in this paper. Nevertheless, to demonstrate the trend in food import volumes pre-trade liberalisation and post-trade liberalisation, 1980 food import volume data is presented in the results section as it reflects total food import volumes before any tariff duties were introduced and before trade liberalisation took effect.

## Results

### Total food import volumes

**Fig. 1:**
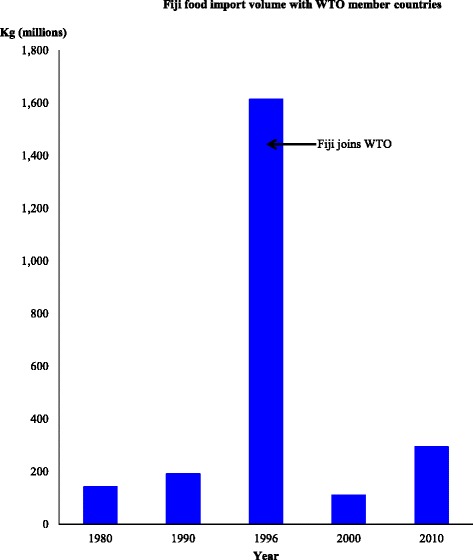
Fiji total import volume with WTO member countries for selected years. *Source:* Data extracted from Fiji Bureau of Statistics (1980, 1990, 1996, 2000); Fiji Revenue and Customs Authority ASYCUDA (2010) [21]

The total volume of food imports into Fiji is from 112 WTO member countries. As shown in Fig. 1, there were high and increasing levels of food import volumes from WTO-member countries and Fiji between the period 1980 and 1996, a sharp decline in the year 2000 and an increase in food import volumes for 2010. The decline in food import volumes in the year 2000 is likely the result of both the re-introduction and implementation of import substitutions measures in the late 1990s [10] and the third military coup in May of that year.

At accession to WTO in 1996, all agricultural products (including food products) had tariffs set at either 0 per cent, 3 per cent, 10 per cent, 20 per cent or at the maximum *ad valorem* tariff set at 27 per cent. From 2004, a revised tariff structure was implemented and consisted of four tariff bands (0 per cent, 5 per cent, 15 per cent and 27 per cent) but new changes introduced during the 2009 Fiji Government Budget has increased all the tariffs in the 27 per cent band to 32 per cent [20]. The variations in tariff rates applied to focus foods are shown in Tables 4 and 5.

**Table 4:** Tariff rates for healthy focus foods

Healthy focus food	1990%	1996%	2000%	2010%
	Applied	Applied	Applied	Applied
Fresh Fruits				
Citrus*	22.5	10	3	15
Fresh apples	5	10	3	5
Fresh grapes	5	10	3	5
Fresh tomatoes*	25	10	3	32
Fresh Vegetables Including Staple Root Crops				
Garlic*	0	0	0	0
Onions*	0	0	0	0
Leeks and other alliaceous vegetables*	10	10	3	5
Cauliflowers and broccoli	10	10	3	32
Other lettuce, other vegetables*	10	10	3	32
Carrots and turnips*	10	10	3	32
Potatoes*	10	22.5	3	32
Celery*	10	10	3	5
Pulses, Nuts and Seeds				
Dried leguminous vegetables, split lentils, chickpeas and kidney beans*	10	10	3	5
Staple, whole-grain cereals				
Rice (brown)*	0	40	40	15
Rolled Oats or Oat meal	25	22.5	3	5
Healthy breakfast cereals	25	22.5	3	5

*Source:* Pacific Islands Legal Information Institute (PACLII) Database – www.paclii.org [34]; WTO Tariff Download Facility – http://tariffdata.wto.org/ [35]; McGregor, A. (2003) [19]

Note: Tariff data for specific food categories in 1980 not available

* Focus foods marked with an asterix are grown locally as well

**Table 5:** Tariff rates for less healthy focus foods

Less Healthy Focus Food	1990%	1996%	2000%	2010%
	Applied	Applied	Applied	Applied
Edible Oil & Spreads				
Palm Oil	25	22.5	20	15
Corn Oil	25	22.5	20	15
Hydrogenated fats, lard, dripping, TINCOL, Etc.	25	22.5	20	15
Margarine	25	22.5	20	15
Butter	25	22.5	27	5 32
Fatty Meat Products				
Pork sausage	25	22.5	27	15
Chicken nuggets/Patties	25	22.5	27	32
Beef Patties	25	22.5	27	32
Canned fish (Processed)	25	22.5	27	15
Canned meat (Processed meat such as spam, corned beef, corned mutton)	25	22.5	27	32
High-Fat Processed Dairy Products				
Processed cheese	25	22.5	27	15
Fruit based/Flavoured yoghurt	5	22.5	10	15
Ice-cream and edible ices	10	22.5	10	32
Energy-Dense Beverages				
Cordial/Juices	25	22.5	27	15
Soft drink	25	22.5	27	15
Electrolyte drinks	25	22.5	27	15
Sugar				
Sugar & other caloric sweeteners	25	22.5	27	32
Savoury ready-to-eat Snacks				
Crisps and snacks	22.5	22.5	27	32
Noodles	10	22.5	27	32
Sweet Snacks				
Confectionary	25	22.5	10 27	15 32
Sweet Biscuits	22.5	22.5	27	32
Sweet Packaged Breakfast Cereals	25	22.5	27	32
White Rice	0	40	40	15

*Source:* Pacific Islands Legal Information Institute (PACLII) Database – www.paclii.org [34]; WTO Tariff Download Facility – http://tariffdata.wto.org/ [35]; McGregor, A. (2003) [19]

Note: Tariff data for specific food categories in 1980 not available

As part of its WTO obligation, Fiji is not only required to progressively reduce its tariff levels on imported products, but it is also required to narrow its dispersion of tariffs in accordance with Article XXVIII *bis* on tariff negotiations. Table 4 highlights that reduction of duties only occurred in 4 of the 15 healthy foods categories in 1990 to 1996. These were for citrus, tomatoes, rolled oats, and healthy breakfast cereals. Tariff rates for all other healthy foods categories remained the same except for potatoes which had a 12.5 per cent increase and brown rice which had a 40 per cent increase. Furthermore, Table 4 indicates that tariff rates were only reduced for brown rice from 2000 to 2010 while all other healthy foods tariffs increased from 2000 to 2010.

In terms of less healthy foods, Table 5 indicates that 6 of the 23 less healthy focus foods did not undergo progressive liberalisation from 1990 to 1996. These were yoghurt, ice-cream and edible ices, crisps and snacks, noodles, sweet biscuits and sweet packaged breakfast cereals. While tariff reductions occurred for other less healthy focus foods highlighted in Table 5, these reductions were small and averaged around 2.5 percentage points. This is in contrast to tariff reductions occurring for the years 2000 and 2010, which were largely in the range of 5 to 22 percentage points. For these specific years, Table 5 also indicates that tariff rates increased for 10 of the 23 less healthy food categories listed. While the increasing tariff levels are not compatible with WTO obligations concerning tariff level reduction, “the flexible use of tariff protection to assist in economic development” outlined in Article XXVIII *bis* has been applied by Fiji as part of its government’s efforts to guard its revenue base, protect its local manufacturing industry and prevent revenue evasion from importers.

### Focus food import volumes

Fig.s 2, 3, 4, 5 illustrate the changes in import volumes for the various focus foods over the selected years. The country of origin of the food products mirrored the country with which Fiji had traditional economic and political ties, for example, Australia, New Zealand, Japan, United States and the European Union, and those with which Fiji has more recently had growing economic and political ties with namely Indonesia, Malaysia, China, Hong Kong, Thailand, Singapore and the Philippines. Overall, the selected healthy focus foods came from 15 major trading partners while less healthy focus foods were from 35 major trading partners as far as South and North America, Europe, Middle East, Asia and Oceania.

**Fig. 2:**
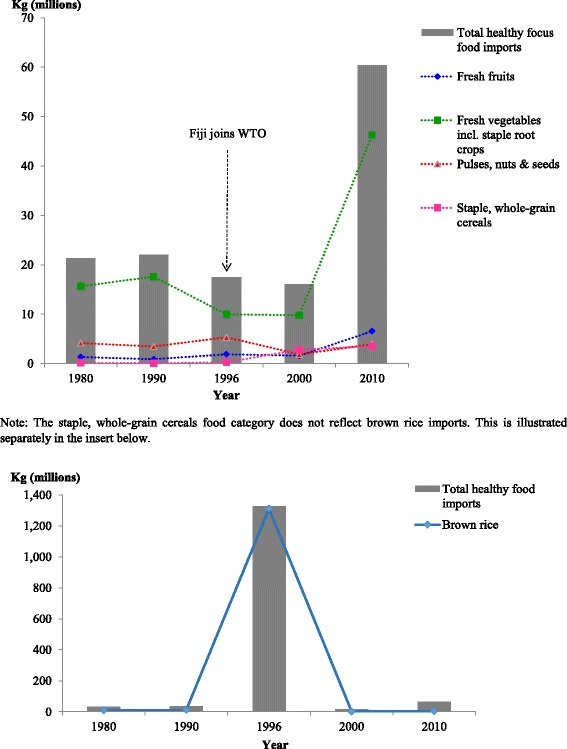
Volume of healthy food imports to Fiji, over the selected years from major WTO importing countries. *Source:* Data extracted from Fiji Bureau of Statistics (1980, 1990, 1996, 2000); Fiji Revenue and Customs Authority ASYCUDA (2010) [21]

#### Healthy food categories import volumes

The changes in volume of selected healthy food imports to Fiji varied in the selected time period, from 1980 to 2010. The insert below Fig. 2 shows a dramatic increase in brown rice imports in the year 1996 under the staple, whole-grain cereals food category. This was followed by a dramatic decrease in 2000 and a slight increase in 2010. This is likely to correspond to the tariff reforms and removal of import licensing controls that Fiji was undertaking as a result of its accession to the WTO and its conformity to these provisions – see section on Tariffs. In terms of other healthy food categories, Fig. 2 also shows an increase in fresh fruits and fresh vegetables including staple root crops in 2000 and 2010. Staple, whole-grain cereals and pulses, nuts and seeds also increased in 2000 but then decreased in 2010.

**Fig. 3:**
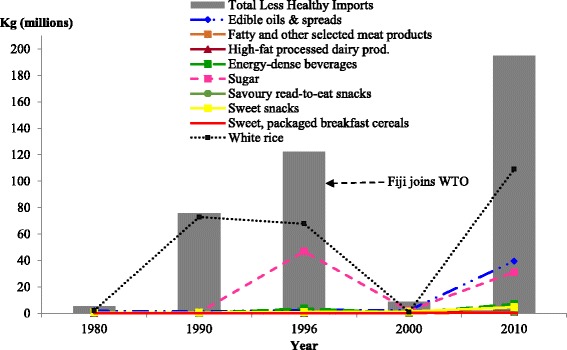
Volume of select less healthy food imports to Fiji, over the selected years from major WTO importing countries. *Source:* Data extracted from Fiji Bureau of Statistics (1980, 1990, 1996 and 2000); Fiji Revenue and Customs Authority ASYCUDA (2010) [21]

Besides GATT 1994, another WTO agreement that has impacted the trend of food import volumes into Fiji is the WTO Agreement on the Application of SPS. According to the relevant provisions of paragraphs 1 through 3 of Article 3 in this agreement, “Members shall base their sanitary and phytosanitary measures on international standards, guidelines or recommendations…and conform to these.” In complying with these provisions and to ensure compatibility with the SPS Agreement, Fiji changed its SPS policy from zero to minimal risk by the time it became a member of the WTO in 1996 and this further opened up the market for various agricultural products including the importation of fruits, vegetables, foodstuffs and poultry [25]. Fiji’s policy switch from zero-risk to minimal risk regulations governed by its SPS commitments relaxed tight controls on imports and the impact of this policy is evident on the growth of fresh fruit and vegetable imports in 2000 and 2010 (Fig. 2) and other less healthy foods (Fig. 3).

#### Less healthy food categories import volumes

The volume of imports of highly-processed, energy-dense and/or high fat foods increased between 1990 to 1996, with further marked increases from 2000 to 2010 (Fig. 3). In 1989, the removal of import licensing controls on 34 food items including white rice, meat products, snack foods, non-alcoholic beverages (including energy-dense beverages) and sugar opened up the Fiji market for increased imports of these between 1990 and 1996. In 1996, a protective tariff for white rice was set at 40 per cent. Consequently, this corresponded to a slight decrease in volume in 1996 and a more significant decrease in volume in 2000, suggesting a possible time-lag effect. Also, when tariffs were reduced in 2010, volumes for foodstuffs, interestingly, increased.

Table 5 details the tariff rates that apply to the selected less healthy foods in Fig. 3. Between 1990 and 2010 tariff on edible oils and spreads decreased from 25 per cent to 15 per cent, except for butter. Likewise, the tariff on white rice also decreased from 40 per cent to 15 per cent. Fig. 3 shows a marked increase in both fats and oils and white rice in 2010. However, applied tariffs on other selected less healthy foods including ice-cream and edible ices, savoury ready-to-eat snacks, sweet snacks and sweet packaged breakfast cereals have increased. Despite this, total food imports in these categories have also increased from 2000 to 2010.

**Fig. 4:**
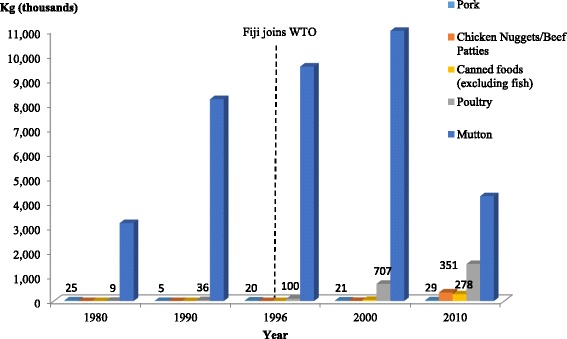
Selected fatty and other meat imports into Fiji for the selected years from WTO partner countries. Note: Data collected for chicken nuggets/beef patties and canned foods were not available between 1980 and 1996. Data on poultry meat and sheep meat was extracted from the FAO TradeStat Database [28]

Table 5 also highlights the relapses in tariff levels for selected meats including mutton, poultry, pork sausage, chicken nuggets and patties, beef patties and canned meat. Import licensing controls were removed from all these foods in 1989 [19] and replaced with applied tariffs of 25 per cent in 1990 reduced to 22.5 per cent in 1996. Fig. 4 shows an increase in poultry and mutton imports during this period. In 2000, mutton and poultry imports continued to increase despite the increased tariff of 27 per cent on all meat products. From 1997, Fiji applied an import licensing restriction on poultry imports from the USA which contributed to the limited import volume of poultry to Fiji in 2000. In 2010, the tariff on all meat products was increased to 32 per cent. Mutton imports declined in 2010 and the import volume of poultry, canned foods and chicken nuggets/beef patties all increased. In addition to the 32 per cent tariff applied on mutton, there was also a 15-year ban on mutton flaps imposed in 2000 [10]. Both of these likely contributed to the decline in mutton imports in 2010. The increase in chicken nuggets and beef patties can be attributed to the increase in fast food restaurants through foreign direct investment (FDI) established in Fiji from 1996 onwards (see discussion later in the paper and Table 6).

**Table 6:** Type and parent company of foreign-owned food corporations operating in Fiji, 2015

Parent Country	Parent Company	TFC	Type of business	Trade Activity	Year of Investment
Australia	Buderim Ginger Limited	Freshpac Ginger (Fiji) Limited	Food preparations	Exports	N/A
Australia	Nestle Australia LTD	Nestle (Fiji) Limited	Food preparation; Candy and other confectionary products; Managing consultancy service	Imports and Exports	1984, expanded 1991
France	W B Finance et Partenaires	Atys (Fiji) Limited	Frozen Fruits and Vegetables; Canned specialities	Imports	N/A
Australia	Goodman Fielder Limited	Crest Chicken Limited	Prepared feeds; Poultry hatcheries	N/A	N/A
Australia	Coca-Cola Amatil Limited	Coca-Cola Amatil (Fiji) Limited	Bottled and canned soft drinks	Imports	1995
Australia	Goodman Fielder Limited	Goodman Fielder (Fiji) Limited	Flavoring extracts and syrups; Ice cream and frozen desserts; Frozen specialties; Prepared feeds	Imports and Exports	N/A
USA	McDonalds	McDonalds Fiji	Fast food restaurant	Food & Beverage Service	1996
USA	Kazi Foods Corporation Fiji	Kentucky Fried Chicken	Fast food restaurant	Food & Beverage Service	2002 (Closed down in 2011)
Australia	Chicken Express Systems P/L	Chicken Express	Fast food restaurant	Food & Beverage Service	2009 Expanded to 10 outlets across Fiji
Australia		Pizza Hut	Fast food restaurant	Food & Beverage Service	2006
Australia	Phoenix Foods Limited	Eagle Boys Pizza	Fast food restaurant	Food & Beverage Service	2015 (opened in 2004, closed down and reopened again)
USA	Motibhai Group	Burger King	Fast food restaurant	Food & Beverage Service	2014 (opened in 2015, now expanded to 2 outlets in Nadi)

*Source:* Data extracted from the International Trade Centre (ITC) database (http://www.intracen.org/) [36]; Fiji TV (http://fijione.tv/burger-king-now-in-fiji/) [37]; Motibhai Group Fiji (http://www.motibhai.com/News-Events/Franchise-boost-for-Company.aspx) [38]; Thow et al., 2011 [10]

*N/A* data not available

**Fig. 5:**
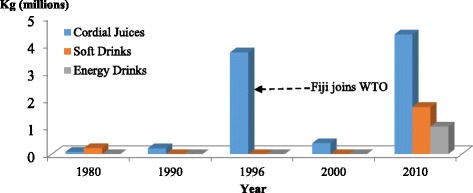
Cordial juices, soft drinks and energy drink imports into Fiji for the selected years from WTO partner countries. *Source:* Data on soft drinks was not available in the 1990 to 2000 data sets. Data on energy drinks was not available in the 1980 to 2000 data sets. Data extracted from Fiji Bureau of Statistics (1980, 1990, 1996 and 2000); Fiji Revenue and Customs Authority ASYCUDA (2010) [21]

As previously mentioned, there were relapses in tariff levels for energy-dense beverages. As Table 5 shows, import tariff rates for cordial juices, soft drinks and electrolyte/energy drinks were reduced from 25 per cent in 1990 to 22.5 per cent in 1996, increased to 27 per cent in 2000 and then reduced to 15 per cent in 2010. In line with the tariff information provided here, Fig. 5 depicts a similar trend for cordial juice import volumes with an increase in 1980, 1990 and 1996, followed by a marked decline in 2000. However, there was not a marked decline in imports of other beverages despite tariff reductions. In 2010, there was a spiked increase again in the import volumes of cordial juices. There was also an increase in import volumes of soft drinks and energy drinks in 2010.

### Trade in services and foreign direct investment

Following its accession to WTO in 1996, Fiji made specific GATS commitments for the tourism and travel-related services sector via which the food industry benefits. In accordance with the GATT Article III on national treatment, and paragraphs 1 through to 3 in Article XVII of GATS, Fiji has applied no limitations on market access and no limitations on national treatment for foreign investors. Only normal government approval and registration is required for foreign investors. Fiji has no bilateral investment treaties.

#### Type and country of origin of all foreign-owned TFCs operating in country

There is no complete dataset available that accounts for all the trans-national food corporations (TFCs) that have entered Fiji and are currently operating; nor is there any available FDI data specific to their investment in domestic food production, processing retail and advertising sectors. Based on the data that are available, there are 11 TFCs identified as operating in Fiji in 2015 (Table 6). These TFCs are mainly associated with food preparation processing and the production of flavouring extracts and syrups, ice-cream and frozen desserts, frozen fruits, meats and vegetables, canned fruits and vegetables, candy and confectionary products and bottled and canned soft drinks. The production of these are both for local consumption and for export. As indicated in Table 6, the fast food industry is also an expanding industry with the introduction of fast food franchisees namely McDonalds, Burger King, Pizza Hut, Kentucky Fried Chicken and Eagle Boys Pizza around Fiji.

## Discussion

Fiji’s WTO membership and associated economic and agricultural policy changes, as well as domestic political issues such as military coups have contributed to increased availability of the diverse range of imported foods described in this paper – fresh fruit and vegetables; whole-grain and refined cereals; fats and oils; meat; processed dairy products; energy-dense beverages; and processed and packaged foods. In terms of the study aim and based on the results presented in this paper, the association between Fiji’s commitments under WTO trade agreements and food import volume trends is complex. While liberalisation resulting from Fiji’s WTO commitments have changed the food availability and nutrition quality of Fiji’s food environment, there are several important and interesting caveats to note. The main points for discussion in this section focus on a series of liberalisation processes based on Fiji’s WTO commitments accompanying the changes in food imports and how these have contributed to shaping Fiji’s food environment, in both positive and negatives ways by increasing both healthy and less healthy food imports.

### Structural adjustment reforms: Import licensing controls and the use of tariffs

Fiji’s move towards free market reforms from 1986 onwards and its policies of export promotion in the 1980s favoured a less trade-restrictive environment accompanying changes in food imports to Fiji. This was coupled with Fiji’s WTO membership and WTO commitments to trade liberalisation which included the phasing out of import licensing controls on imports including food items such as white rice, meat products, snack foods, non-alcoholic foods and sugar. The removal of import licensing controls on all agricultural products (including food items) appears to have contributed to an increase in fresh fruits, pulses, nuts and seeds, staple-whole grain cereals, including brown rice, (as shown in Fig. 2) and an increase in sugar, energy-dense beverages, sweet snacks and savoury ready-to-eat snacks, shown in Fig. 3.

Fiji’s commitment to tariff reductions has also played a role in shaping the food environment by increasing both healthy and unhealthy food imports into Fiji. As a signatory to GATT 1994 and in accordance with GATT Article I (1) regarding the application of customs duties and charges of any kind with respect to Article XXVIII *bis*, Article XXIV (8), Article XI (1), Article XIII (1), and Article IX (1) on the importation or exportation of products, Fiji like any other WTO country-member is bound to gradually eliminate tariff and non-tariff barriers to trade. As of 1994, Fiji has been bound to these liberalising commitments in relation to its trade activity with 127 trading partners now increased to 161 trading partners at the end of 2015. As the analysis in this paper highlights, Fiji’s tariff reforms and reduction commitments can have positive effects on the food supply, such as the increase in supply of fresh fruits, fresh vegetables and staple root crops - despite tariff reductions or increases during periods of import substitution and agricultural investment. The tariff reforms have also had negative effects on the food supply, with the influx of less healthy food imports. However in both instances, the relationship is not straightforward, and depends on a range of considerations as summarised below.

The spike in some of the food import volumes to Fiji from a variety of countries in 1990 and 1996, as illustrated in Figs. 2 and 3, corresponds with the removal of import licensing controls from agricultural products and tariff reforms that Fiji was undertaking as a result of its accession to the WTO and its conformity to these provisions. For example, the volume of white rice imports spiked in 1990 and began to decline in 1996 after the introduction of a high tariff rate (of 40%). However, in the case of brown rice there was still a significant trade into Fiji in the same year despite the 40 per cent tariff rate placed on it. While there seems to be a different observed time-lag effect for brown rice and white rice, it seems that the rice import volumes are also responding to other factors, not just to tariffs. For some food categories such as fatty meats, high-fat processed dairy products, energy-dense beverages, sugar and savoury ready-to-eat snacks, the tariff reductions and increases appear to have corresponded directly to the total percentage import volumes of these foods. In the years where the percentage import volumes of these foods increased, despite increased tariff rates placed on specific foods in some of these categories, the size of tariff reductions were relatively small (averaging between 2.5 to 5 percentage points). Based on Fig. 2 and Fig. 3, there were also some instances where decreases in tariffs were not matched by increased food import volumes. Where this is evident, the magnitude of tariff reductions was also relatively small. There appears to have been a dose-response effect in terms of the magnitude of tariff reductions and the increase in food imports in 2000 and 2010. This is evident in the fresh fruits category (Fig. 2) and the edible oils and spreads category (Fig. 3). In the case of fresh vegetables including staple root crops, increase in tariff rates from 3 percentage points to 32 percentage points for the majority of foods listed in Table 4 were not matched by decreased food import volumes. The continued increase in percentage food import volume in the year 2010 is likely the result of a category 4 cyclone that struck the Fiji islands in March 2010 in which the agriculture sector suffered major damages. Moreover, the combination of backsliding in tariff levels in the year 2000 and 2010, coupled with the political instability resulting from a military coup in the year 2000, a major policy reversal in the late 1990s back towards government-led agricultural development and again in 2010, which have resulted in the implementation of import substitution measures [10], are likely to have contributed to a decline in total food import volumes in these years.

### Fiji’s WTO commitments: WTO agreements on technical barriers to trade (TBT), import licensing procedures and the application of sanitary and phytosanitary measures (SPS)

Globally, the consumption of processed foods and sugar-sweetened beverages has been shown to correlate with increased obesity and NCD risk. To regulate the sale and availability of these in the food environment, the Agreement on Technical Barriers to Trade (TBT) and the Agreement on Import Licensing Procedures provides ground for technical regulations to be prepared, adopted or applied by Members to protect human health and safety. Fiji is yet to maximise the general safeguards in line with these agreements and has only imposed restrictions for a few products in accordance with Articles 2.1, 2.2 and 10 of the TBT related to the treatment of imported products and the appropriate measures that Members should apply. For the Agreement on Import Licensing Procedures, Fiji has imposed restrictions in accordance with paragraphs 4 and 8 of Article 1, and paragraphs 1, 2 and 4 of Article 14 related to the application of licensing requirements for imports. In accordance with Article 10.6 of the TBT, Fiji notified WTO member countries of its pursuit in importing salt with high iodine level content as opposed to salt without iodine. Hence, the importation of salt without iodine is controlled and covered by non-automatic import licensing. Besides salt, margarine, butter and condensed milk are the only other food products covered by non-import licensing. The high import volumes of palm oil and hydrogenated fats as shown in Fig. 3 could be attributed to this non-automatic import licensing. The Ministry of Health in Fiji is the controlling authority for the non-automatic import licenses accorded to these products. Under the Agreement on Import Licensing Procedures and in accordance with paragraphs 1 and 8 of Article 1, Fiji has only applied an import licensing restriction to the importation of chicken products approved by the USDA from the USA which came into effect in 1997. Through this arrangement, chicken imports are limited to 16,000 tonnes per year with a further distribution breakdown of 100 tonnes per province (14 provinces in Fiji) and 100 tonnes per applicant. In cases where the importation of chicken products exceeds the quota, these are not sold but held at the wharf for transit. In addition to this, Fiji applied SPS conditions under the SPS Agreement in 2001 and prohibited the importation of uncooked poultry meat vaccinated against Newcastle Disease from Australia, New Zealand and USA. This was done in accordance with paragraphs 1 to 3 of Article 3 in the SPS. Fig. 4 illustrates how this license control contributed to the limited import volume of poultry to Fiji in 1996 and 2000.

The increase in various healthy and less healthy food imports in 2000 and 2010 also correspond with Fiji’s policy switch from zero-risk to minimal risk regulations governed by its WTO SPS commitments. Although the tariff rates for some healthy food categories, such as fresh fruit and vegetable imports, and less healthy food categories, such as fatty meat products, ice-cream and edible ices, savoury ready-to-eat snacks and sweet snacks increased between 2000 and 2010, the import volumes of these also increased. In addition to the role of tariffs and the size of tariff changes in altering food import volumes, the relaxation of tight controls on imports through this policy switch and the commitments that Fiji has made under various WTO Agreements including the Agreements on TBT, SPS and Import Licensing Procedures are other factors to consider in understanding the association between Fiji’s commitments under the WTO trade agreements and food import volume trends.

### Fiji’s WTO Commitment to foreign direct investment

The encouragement of foreign direct investment through Fiji’s commitments to GATS and GATT 1994 has also increased availability of locally produced food preparations and processed foods and ultimately the consumption of these [10]. While there is no complete dataset on investment by food corporations, this is likely to have grown with overall foreign direct investment, which has increased markedly in Fiji [26]. As presented in Table 6, the major fast food franchisees such as McDonalds, Kentucky Fried Chicken and Burger King and major transnational food companies such as Coca-Cola Amatil (Fiji) Limited and Nestle (Fiji) Limited have shaped the food environment significantly as has been documented in other developing countries [27]. Most of these investment have occurred since economic liberalization in Fiji.

The production of foods by TNCs as presented in Table 6 for both local consumption and for export have important implications on the nutritional quality of the food environment in Fiji. Food preparations including flavouring extracts and syrups for the production of confectionaries, ice creams and edible ices, as well as soft drinks, candy and other confectionary products are now produced locally. These are increasingly consumed as they are readily available in the local food environment and are much cheaper alternatives than imported soft drinks, ice-cream and frozen desserts. Noodles, in the savoury ready-to-eat snacks category, is one food that was previously documented to be increasingly consumed in Fiji and is among the top 12 common energy foods eaten in both urban and rural areas. Noodles are directly affected by investment [10]. Despite the increase in tariff rates for noodles from 2000 to 2010, the import volume of noodles continued to increase in 2010. This is likely associated with rising availability as the result of domestic production (Nestle invested in a factory producing noodle which commenced in 1984) [10] but also from the opening up of the market to other major noodle exporting WTO member countries such as Indonesia, China, Malaysia, Thailand, Singapore and the Philippines. In terms of export, there are certain categories of healthy and less healthy food inputs that are used for processing and then exported to other Pacific Island Countries. For example, healthier inputs for the processing of frozen fruit and vegetables and canned specialities are brought in for food processing and then exported out to neighbouring countries including Kiribati and Tuvalu. In this instance, liberalization is strongly associated with a poor quality local food environment. Similarly, there are also less healthy food inputs imported for processing and export including flavouring extracts of syrups and food preparations to manufacture edible ices, frozen desserts and bottled and canned soft drinks. While it is beyond the scope of this study to assess the proportion of food inputs imported (both healthy and less healthy) used for processing and exported again to other neighbouring Pacific island countries, the rising availability of less healthy foods such as ice-cream, frozen desserts and soft drinks for local consumption due to domestic production is most greatly associated with foreign direct investment.

### Trade liberalisation and food import volume trends in Fiji

The effect of Fiji’s WTO commitments and trade policy changes appears to have been unconstrained given the relatively limited protection and safeguard measures undertaken to protect public health and to regulate the increased availability of processed foods. It is possible, however, for Fiji to intervene through several WTO agreements that govern the use of safeguard measures for reasons such as protection of public health. However, extensive procedural requirements and conditions make these mechanisms difficult to use [19] and as such, Fiji has only undertaken safeguard measures for a handful of food products including salt, margarine, butter and poultry meat. The analysis in this paper suggests that commitments to trade agreements can have both positive and negative effects on the food environment by opening up the market to a diverse range of food products during periods of progressive tariff reduction and the subsequent removal of non-tariff barriers, however the change in food import volumes also depends on other considerations as highlighted. In Fiji, the government banned the sale of lamb/mutton flaps in the year 2000 using the concept of non-discrimination under the principle of ‘national treatment’ whereby countries should not treat imports less favourably than the same or similar domestically produced goods once they have passed Custom [6]. As such, Fiji’s sales ban as an approach to food policy to reduce unhealthy imports can be used to improve the food supply in a trade compliant way.

The impacts of trade agreements on food environments intersect with a variety of other pathways including globalisation patterns of development and other socio-demographic changes brought about through increasing urbanisation, the growth of a monetized economy and changing work and leisure patterns [10]. Though, it has been difficult to draw firm conclusions on the evidence linking the impact of trade agreements on food environments within and across countries as opposed to the other socio-demographic changes mentioned. While this study provides evidence for the effect of specific provisions of WTO agreements on the volume of food imports entering Fiji, it also indicates that more monitoring work needs to be carried out through the use of the ‘expanded’ and ‘optimal’ approaches of the INFORMAS trade protocol to better understand and validate the relationship between trade agreements and their role in shaping national food environments.

### Study limitations

This study used a variety of complementary data sources to assess changes in food import volumes and the healthiness of Fiji’s food environment in response to trade liberalisation and foreign direct investment committed under Fiji’s membership to the WTO. Given the limited data available the study cannot demonstrate causality nor can we effectively estimate the importance of trade agreement provisions in driving change in nutrition quality and the healthiness of Fiji’s food environment. Due to the limited availability of data and resources available, we only focussed on two domains of the INFORMAS framework. We were also unable to consider the effect of the WTO Agreements which Fiji is party to under the ‘domains’ of domestic protections and supports and policy space and governance, on the healthiness of Fiji’s food environment. The study was also limited by gaps in available data; in particular, data relating to tariff rates, FDI investment, schedules of specific commitments and government procurement. The calculation of tariff-rate quotas for focus food categories and the calculation of tariff-differentials between ‘healthy’ and ‘less healthy’ focus food categories was lacking. There was also no monetary data readily available for FDI investment in transnational food corporations. In terms of domestic protections and support, the schedules of specific commitments pertaining to agricultural subsidies is not updated and data on government procurement is not available limiting us from carrying out any assessment under the fourth domain. The reliability of data was also an issue; in particular, data relating to classifications of foods, partner countries, the volume of the products and quantity of measurement.

## Conclusion

The analysis presented in this paper shows that Fiji’s commitments to WTO Agreements do play an important role in shaping the food environment by increasing both healthy and less healthy food imports. The monitoring aspect of this research suggests that the development of a systematic approach to monitoring the impact of trade agreements on the food supply at a country level is critical for developing appropriate and targeted interventions to improve diets and health. This would enable national health interventions to both identify areas of concern, and to ensure that interventions take into account the trade context.

While the changes in food import volumes coming to Fiji in the selected years correspond to Fiji’s schedule of specific commitments under the WTO Agreements, the complexity of the association between tariff reductions, other geo-political considerations and the use of non-tariff barriers and domestic policy controls have also contributed to the changes in food availability and nutritional quality in Fiji’s food environment. This reinforces the need to monitor the impacts of trade agreements to address food supply factors at the national level through trade policy commitments that ultimately contribute to the availability and nutritional quality of the food supply in national food environments.

## Footnotes

1
http://tariffdata.wto.org/

2
http://www.intracen.org/
